# Reduced low-density lipoprotein cholesterol levels are associated with increased risk of gestational diabetes mellitus in Chinese women

**DOI:** 10.1038/s41598-025-91258-8

**Published:** 2025-02-22

**Authors:** Shuai Zeng, Qingqing Liu, Yujie Wu, Huai Bai, Ping Fan, Xinghui Liu

**Affiliations:** 1 Department of Obstetrics and Gynecology, West China Second University Hospital, Sichuan University, Chengdu, Sichuan China; 2 Laboratory of Genetic Disease and Perinatal Medicine/Laboratory of the Key Perinatal Disease, Key Laboratory of Birth Defects and Related Disease of Women and Children, Ministry of Education, West China Second University Hospital, Sichuan University, Chengdu, Sichuan China

**Keywords:** Low-density lipoprotein cholesterol, Gestational diabetes mellitus, Lipid profile, Gestational diabetes, Risk factors, Lipoproteins

## Abstract

Lipid levels in women with gestational diabetes mellitus (GDM) have been extensively studied, whether low-density lipoprotein cholesterol (LDL-C) is a risk factor for GDM development remains unclear. This study aimed to investigate the correlation between serum LDL-C levels and the risk of GDM. A case–control study was conducted. Glycolipid metabolic and oxidative stress indicators were measured in 696 women with GDM and 1048 healthy pregnant women. Serum LDL-C levels were significantly lower in the GDM group than in the control group (*P* < 0.001). Subgroup analysis indicated that reduced LDL-C levels were associated with an increased risk of GDM after adjusting for differences in maternal age, pre-pregnancy body mass index (BMI), gestational age at sampling, fasting glucose and insulin levels, and homeostatic model assessment of insulin resistance (odds ratio [OR] 1.372, 95% confidence interval [CI] 1.050–1.794, *P* = 0.021 for medium-LDL-C subgroup; OR 1.672, 95% CI 1.219–2.294, *P* = 0.001 for low-LDL-C subgroup). The risk of GDM decreased by 17.6% per 1 mmol/L increase in LDL-C level (OR 0.824, 95% CI 0.733–0.926, *P* = 0.001). Furthermore, apolipoprotein (apo) A1 and high-density lipoprotein cholesterol (HDL-C) levels were lower, whereas pre-pregnancy and delivery BMI, triglyceride (TG)/HDL-C ratios, and second-trimester fasting glucose levels were higher in the low-LDL-C GDM subgroup than those in the high- and/or medium-LDL-C GDM subgroups (*P* < 0.05). ApoA1 and HDL-C levels were lower but TG/HDL-C ratios were higher in the medium-LDL-C GDM subgroup than those in the high-LDL-C GDM subgroup (*P* < 0.05). We concluded that reduced LDL-C levels were associated with an elevated risk of GDM in the study population. Low LDL-C levels correlated with increased BMI and unfavorable TG, HDL, and glucose metabolism.

## Introduction

Gestational diabetes mellitus (GDM) is a prevalent pregnancy complication characterized by impaired glucose tolerance^1^. The incidence of GDM differs among populations with an estimated 14.8% prevalence in pregnant Chinese women^2^. GDM has been associated with diverse maternal and neonatal health risks that can have short- or long-term effects, including preterm birth, preeclampsia, macrosomia, type 2 diabetes mellitus (T2DM), cardiometabolic disease, and obesity^1,3^. Although the pathogenesis of GDM is not entirely understood, increasing evidence indicates various risk factors, including a higher pre-pregnancy body mass index (BMI), advanced maternal age, imbalanced hormone release, increased oxidative stress, persistent inflammation, dyslipidemia, dysfunction of pancreatic β-cells, genetic variants, and the use of assisted reproduction technology^1,4–7^.

Gestation is a stress status and promotes the formation of an atherogenic lipid profile in women^6,8,9^. Lipid levels decrease in the first two months of pregnancy and then steadily increase during gestation to meet the nutritional needs of fetal growth and development^10^; for example, cholesterol is important for embryo implantation, uteroplacental vascularization, and steroid hormone production^11,12^. The changes in maternal lipid metabolism are closely related to the role of hormones and onset of insulin resistance during gestation^13,14^. Increased levels of estrogen, progesterone, and insulin facilitate lipid production and storage during early pregnancy^10,13,14^. As gestation progresses, the secretion of placental hormones, elevated estrogen levels, and insulin resistance promote lipolysis, stimulate the synthesis and secretion of hepatic lipids, and increase circulating lipoprotein levels^10,13^. However, dyslipidemia significantly influences the development of GDM^9,15–18^. In a meta-analysis of 28,232 individuals with GDM and 69,648 control women, women with GDM had significantly higher levels of total cholesterol (TC), triglyceride (TG), very low-density lipoprotein cholesterol (VLDL-C), and low-density lipoprotein cholesterol (LDL-C), while their high-density lipoprotein cholesterol (HDL-C) levels were lower than those in control women^9^. Abnormal TG, TC, and LDL-C levels during gestation, especially in the mid-trimester, are associated with an elevated risk of abnormal postpartum glucose metabolism in women with GDM^16–18^.

Our previous study unexpectedly indicated that serum LDL-C levels prior to delivery were significantly lower in women with GDM compared with those with uncomplicated pregnancies^4,5^. However, the precise relationship between serum LDL-C levels and the risk of developing GDM remains unclear. In this study, we sought to elucidate the relationship between LDL-C levels and the risk of GDM and to evaluate the effect of various LDL-C levels on a range of clinical, glycolipid metabolic, and oxidative stress indicators in a Southwest Chinese population.

## Results

### Clinical and biochemical characteristics of participants

As illustrated in Table 1, pre-pregnancy BMI and gestational age at sampling were higher in the GDM group than those in the control group. Of the 696 women with GDM, 78 were treated with insulin and the remaining were managed with dietary and exercise modifications.

**Table 1:** Clinical, metabolic, and oxidative stress parameters of women with and without GDM.

	Controls (*n* = 1048)	GDM (*n* = 696)	*P*	*P*^a^
Clinical characteristics				
Age (year)	35.49 ± 3.74	35.45 ± 4.09	0.822	
Pre-pregnancy BMI (kg/m^2^)	21.22 ± 2.68	22.24 ± 2.92	< 0.001	
Delivery BMI (kg/m^2^)	26.72 ± 2.70	26.80 ± 3.17	0.618	
Gestational age at sampling (wk)	35.90 ± 3.55	36.81 ± 3.29	< 0.001	
Gestational age (wk)	39.27 ± 0.84	38.92 ± 1.79	< 0.001	< 0.001
Pregnancy weight gain (kg)	14.05 ± 4.34	11.47 ± 4.26	< 0.001	< 0.001
SBP (mmHg)	115.13 ± 10.11	115.63 ± 11.86	0.089	0.091
DBP (mmHg)	72.07 ± 7.94	72.64 ± 9.11	0.083	0.126
OGTT-fasting Glu (mmol/L)^b^	4.43 ± 0.29	4.89 ± 0.54	< 0.001	< 0.001
OGTT-1 h Glu (mmol/L)^b^	7.47 ± 1.29	9.88 ± 1.41	< 0.001	< 0.001
OGTT-2 h Glu (mmol/L)^b^	6.55 ± 1.02	8.69 ± 1.36	< 0.001	< 0.001
Neonatal birth height (cm)	49.86 ± 1.88	49.64 ± 1.82	0.016	0.001
Neonatal birth weight (g)	3379.30 ± 370.21	3343.39 ± 439.48	0.077	< 0.001
Macrosomia % (n)	4.0 (42)	5.6 (39)	0.121	
Insulin treatment (n)	0	78		
Metabolic parameters				
Fasting Glu (mmol/L)	4.35 ± 0.42	4.62 ± 0.72	< 0.001	< 0.001^c^
Fasting Ins (pmol/L)	72.30 ± 35.98	102.99 ± 124.59	< 0.001	< 0.001^c^
HOMA-IR	2.04 ± 1.09	3.35 ± 5.52	< 0.001	< 0.001^c^
TG (mmol/L)	3.64 ± 1.41	3.90 ± 1.69	0.001	0.068
TC (mmol/L)	6.08 ± 1.09	5.96 ± 1.10	0.025	0.151
HDL-C (mmol/L)	1.99 ± 0.41	1.97 ± 0.44	0.363	0.500
LDL-C (mmol/L)	3.19 ± 0.99	2.97 ± 0.97	< 0.001	0.001
TG/HDL-C	1.92 ± 0.88	2.10 ± 1.12	< 0.001	0.031
non-HDL-C (mmol/L)	4.09 ± 0.95	3.99 ± 0.91	0.028	0.169
AI	2.13 ± 0.59	2.10 ± 0.56	0.298	0.506
ApoA1 (g/L)	2.37 ± 0.43	2.30 ± 0.41	0.001	0.002
ApoB (g/L)	1.15 ± 0.26	1.15 ± 0.26	0.854	0.736
ApoB/apoA1	0.50 ± 0.14	0.51 ± 0.13	0.062	0.041
Oxidative stress parameters^d^				
TOS (µmol H_2_O_2_ Equiv./L)	21.08 ± 7.00	25.79 ± 10.48	< 0.001	< 0.001
TAC (mmol Trolox Equiv./L)	1.11 ± 0.19	1.12 ± 0.20	0.311	0.163
OSI	19.41 ± 7.31	23.26 ± 10.08	< 0.001	< 0.001
MDA (µmol/L)	5.37 ± 1.21	5.87 ± 1.42	< 0.001	< 0.001

Values are presented as mean ± SD.

*GDM* gestational diabetes mellitus, *BMI* body mass index, *SBP* systolic blood pressure, *DBP* diastolic blood pressure, *OGTT* oral glucose tolerance test, *Glu* glucose, *Ins* insulin, *HOMA-IR* homeostatic model assessment of insulin resistance, *TG* triglyceride, *TC* total cholesterol, *HDL-C* high-density lipoprotein cholesterol, *LDL-C* low-density lipoprotein cholesterol, *AI* atherosclerosis index, *ApoA1* apolipoprotein A1, *apoB* apolipoprotein B, *TOS* total oxidant status, *TAC* total antioxidant capacity, *OSI* oxidative stress index, *MDA* malondialdehyde.

^a^ All-parameter comparisons were corrected for differences in age, pre-pregnancy BMI, gestational age at sampling, fasting Ins, fasting Glu, and HOMA-IR between the two groups, except for age, BMIs, gestational age at sampling.

^b^ Plasma glucose levels during OGTT between 24 and 28 weeks of gestation.

^c^ Comparisons of fasting Ins, fasting Glu, and HOMA-IR were corrected for differences in age, pre-pregnancy BMI, and gestational age at sampling between the two groups.

^d^ Controls (*n* = 819), GDM (*n* = 571).

Since maternal pre-pregnancy BMI, gestational age at sampling, and the use of insulin were different between the GDM and control groups, differences could bias the comparisons of oxidative stress and metabolic profile. Therefore, the following comparisons of all relevant variables between the groups/subgroups were corrected for differences in age, pre-pregnancy BMI, gestational age at sampling, fasting insulin, fasting glucose, and homeostatic model assessment of insulin resistance (HOMA-IR).

Pregnancy weight gain, gestational age, neonatal birth weight and height, apolipoprotein (apo) A1, and LDL-C levels were significantly lower, while fasting-, 1 h-, and 2 h-glucose levels in the OGTT between 24 and 28 weeks of gestation, apoB/apoA1, TG/HDL-C, fasting glucose and insulin, HOMA-IR, malondialdehyde (MDA), total oxidant status (TOS), and oxidative stress index (OSI) were significantly higher in the GDM group than those in the control group (*P* < 0.05).

### Frequencies of different levels of LDL-C in women with and without GDM

According to the distribution of LDL-C levels in all the participants, we divided the GDM women and controls into three subgroups based on the first and third quartile values of LDL-C levels: (i) low LDL-C subgroup: ≤ 2.40 mmol/L; (ii) medium LDL-C subgroup: 2.41–3.67 mmol/L; and (iii) high LDL-C subgroup: ≥ 3.68 mmol/L.

Table 2 indicates that the frequency of women with a low LDL-C level was significantly higher (27.9% vs. 22.1%), and the frequency of women with a high LDL-C level was significantly lower (19.7% vs. 28.9%) in the GDM group than that in the control group (*P* < 0.001). Estimated power of the two-sample proportions test (α = 0.05) was 0.995. Logistic regression analysis demonstrated that decreased LDL-C levels were associated with an elevated risk of GDM when the high-LDL-C subgroup was used as a reference, with pre-pregnancy BMI, gestational age at sampling, maternal age, fasting insulin, fasting glucose, and HOMA-IR as covariates (OR = 1.372, 95% CI: 1.050–1.794, *P* = 0.021 for the medium-LDL-C subgroup; OR = 1.672, 95% CI: 1.219–2.294, *P* = 0.001 for the low-LDL-C subgroup). The risk of GDM decreased by 17.6% per 1 mmol/L increase in serum LDL-C level in a regression model that included all the study participants, with pre-pregnancy BMI, gestational age at sampling, maternal age, fasting insulin, fasting glucose, and HOMA-IR as covariates (OR = 0.824, 95% CI: 0.733–0.926, *P* = 0.001).

**Table 2:** Frequencies of different LDL-C levels in women with and without GDM.

LDL-C level	Controls (*n* = 1048)	GDM (*n* = 696)	OR	95%CI	*P*
≤ 2.40 mmol/L	232 (22.1%)	194 (27.9%)	1.672	1.219–2.294	0.001
2.41–3.67 mmol/L	514 (49.0%)	365 (52.4%)	1.372	1.050–1.794	0.021
≥ 3.68 mmol/L	302 (28.9%)	137 (19.7%)	1.000		

*LDL-C* low-density lipoprotein cholesterol, *GDM* gestational diabetes mellitus, *OR* odds ratio, *CI* confidence interval.

Chi-squared test: χ^*2*^ = 20.450, *P* < 0.001. OR and 95% CI were calculated using a multinomial logistic regression model, with age, pre-pregnancy BMI, gestational age at sampling, fasting insulin, fasting glucose, and HOMA-IR as covariates, and the LDL-C level of ≥ 3.68 mmol/L as the reference category.

### Clinical and biochemical indicators based on different levels of LDL-C in women with and without GDM

As exhibited in Table 3, the subgroup analysis of women with GDM showed that the low-LDL-C subgroup exhibited higher pre-pregnancy BMI, OGTT-fasting glucose levels, and TG/HDL-C ratios but lower apoA1, HDL-C, apoB, TC, non-HDL-C, AI, apoB/apoA1 ratios, and MDA levels than the high- and medium-LDL-C subgroups (*P* < 0.05), was inclined to have decreased OSI than the medium-LDL-C subgroup (*P* = 0.057), and had higher delivery BMI (*P* = 0.005) and tended to have increased TG levels (*P* = 0.082) than the high-LDL-C subgroup. The medium-LDL-C subgroup also displayed lower age, apoA1, HDL-C, apoB, TC, non-HDL-C, AI, and apoB/apoA1 ratios (*P* < 0.05), but higher TG/HDL-C ratios (*P* = 0.011) and tended to have increased fasting insulin levels (*P* = 0.090) than the high-LDL-C subgroup.

**Table 3:** Clinical, metabolic, and oxidative stress parameters according to LDL-C level in women with and without GDM.

	Control group			GDM group		
	High (n = 302)	Medium (n = 514)	Low (n = 232)	High (n = 137)	Medium (n = 365)	Low (n = 194)
Clinical characteristics						
Age (years)	35.39 ± 3.86	35.48 ± 3.74	35.66 ± 3.61	36.04 ± 4.21	35.10 ± 3.97^§^	35.69 ± 4.16
Pre-pregnancy BMI (kg/m^2^)	20.93 ± 2.41	21.21 ± 2.62	21.71 ± 2.73^†,‡^	21.66 ± 2.68	22.13 ± 2.96	22.89 ± 2.92^§,¶^
Delivery BMI (kg/m^2^)	26.47 ± 2.64	26.59 ± 2.69	27.36 ± 2.73^†,‡^	26.24 ± 2.79	26.77 ± 3.40	27.25 ± 2.90^§^
Gestational age at sampling (wk)	36.00 ± 3.54	35.79 ± 3.52	35.99 ± 3.63	37.01 ± 3.26	36.87 ± 3.27	36.56 ± 3.34
Gestational age (wk)	39.28 ± 0.87	39.28 ± 0.83	39.25 ± 0.83	38.95 ± 1.22	39.01 ± 0.88	38.94 ± 1.08
Pregnancy weight gain (kg)	14.19 ± 3.92	13.81 ± 4.03	14.41 ± 5.44	11.65 ± 4.13	11.58 ± 4.26	11.12 ± 4.34
SBP (mmHg)	114.77 ± 9.77	115.19 ± 9.99	115.44 ± 10.84	116.59 ± 10.30	115.45 ± 11.21	116.60 ± 10.42
DBP (mmHg)	71.96 ± 7.21	72.18 ± 7.58	72.27 ± 8.22	73.86 ± 8.11	72.44 ± 8.64	72.84 ± 8.34
OGTT-fasting Glu (mmol/L)^a^	4.42 ± 0.30	4.42 ± 0.30	4.48 ± 0.28^‡^	4.83 ± 0.53	4.86 ± 0.51	5.02 ± 0.57^§,¶^
OGTT-1h Glu (mmol/L)^a^	7.37 ± 1.29	7.45 ± 1.34	7.67 ± 1.18^†^	9.93 ± 1.29	9.87 ± 1.38	9.84 ± 1.57
OGTT-2h Glu (mmol/L)^a^	6.47 ± 1.03	6.53 ± 1.04	6.70 ± 0.94	8.63 ± 1.27	8.64 ± 1.31	8.83 ± 1.49
Neonatal birth height (cm)	49.83 ± 1.84	49.86 ± 2.01	49.90 ± 1.64	49.50 ± 1.76	49.69 ± 1.78	49.65 ± 1.92
Neonatal birth weight (g)	3348.53 ± 379.82	3375.89 ± 364.56	3427.11 ± 366.68	3322.85 ± 414.54	3347.32 ± 422.33	3350.39 ± 487.99
Metabolic parameters						
Fasting Glu (mmol/L)^b^	4.34 ± 0.45	4.36 ± 0.40	4.36 ± 0.43	4.53 ± 0.66	4.63 ± 0.71	4.66 ± 0.79
Fasting Ins (pmol/L)^b^	71.05 ± 38.61	72.09 ± 36.18	74.73 ± 31.18	80.49 ± 58.62	107.79 ± 133.76	110.84 ± 140.98
HOMA-IR^b^	2.00 ± 1.17	2.04 ± 1.11	2.10 ± 0.92	2.44 ± 2.68	3.55 ± 6.08	3.68 ± 5.94
TG (mmol/L)	3.59 ± 1.07	3.51 ± 1.27	4.00 ± 1.93^†,‡^	3.64 ± 1.09	3.89 ± 1.64	4.10 ± 2.05
TC (mmol/L)	7.23 ± 0.77	5.93 ± 0.62^†^	4.91 ± 0.74^†,‡^	7.26 ± 0.83	6.04 ± 0.71^§^	4.90 ± 0.72^§,¶^
HDL-C (mmol/L)	2.11 ± 0.38	1.99 ± 0.39^†^	1.81 ± 0.43^†,‡^	2.22 ± 0.44	2.00 ± 0.39^§^	1.73 ± 0.40^§,¶^
LDL-C (mmol/L)	4.37 ± 0.75	3.03 ± 0.34^†^	1.99 ± 0.32^†,‡^	4.36 ± 0.93	2.98 ± 0.34^§^	1.95 ± 0.33^§,¶^
TG/HDL-C	1.75 ± 0.61	1.83 ± 0.77	2.33 ± 1.21^†,‡^	1.70 ± 0.59	2.02 ± 0.90^§^	2.53 ± 1.55^§,¶^
Non-HDL-C (mmol/L)	5.12 ± 0.72	3.93 ± 0.55^†^	3.10 ± 0.61^†,‡^	5.04 ± 0.68	4.04 ± 0.62^§^	3.16 ± 0.62^§,¶^
AI	2.51 ± 0.60	2.05 ± 0.49^†^	1.81 ± 0.51^†,‡^	2.36 ± 0.53	2.10 ± 0.53^§^	1.92 ± 0.57^§,¶^
ApoA1 (g/L)	2.38 ± 0.46	2.38 ± 0.40	2.32 ± 0.44	2.43 ± 0.52	2.30 ± 0.37^§^	2.20 ± 0.37^§,¶^
ApoB (g/L)	1.44 ± 0.2	1.11 ± 0.14^†^	0.88 ± 0.16^†,‡^	1.46 ± 0.19	1.17 ± 0.16^§^	0.89 ± 0.15^§,¶^
ApoB/apoA1	0.62 ± 0.15	0.48 ± 0.10^†^	0.39 ± 0.10^†,‡^	0.62 ± 0.14	0.52 ± 0.11^§^	0.42 ± 0.10^§,¶^
Oxidative stress parameters^c^						
TOS (μmol H_2_O_2_ Equiv./L)	20.95 ± 6.74	20.87 ± 7.03	21.70 ± 7.29	24.66 ± 9.43	26.61 ± 10.90	25.12 ± 10.41
TAC (mmol Trolox Equiv./L)	1.12 ± 0.19	1.10 ± 0.20	1.10 ± 0.19	1.11 ± 0.21	1.12 ± 0.20	1.12 ± 0.20
OSI	19.21 ± 6.72	19.38 ± 7.63	19.75 ± 7.42	22.97 ± 10.29	24.03 ± 10.38	21.95 ± 9.16
MDA (μmol/L)	5.59 ± 1.19	5.28 ± 1.19^†^	5.31 ± 1.38	6.05 ± 1.23	5.95 ± 1.53	5.57 ± 1.30^§,¶^

Values are presented as mean ± SD.

*LDL-C* low-density lipoprotein cholesterol, *GDM* gestational diabetes mellitus, *BMI* body mass index, *SBP* systolic blood pressure, *DBP* diastolic blood pressure, *OGTT* oral glucose tolerance test, *Glu* glucose, *Ins* insulin, *HOMA-IR* homeostatic model assessment of insulin resistance, *TG* triglyceride, *TC* total cholesterol, *HDL-C* high-density lipoprotein cholesterol, *AI* atherosclerosis index, *ApoA1* apolipoprotein A1, *apoB* apolipoprotein B, *TOS* total oxidant status, *TAC* total antioxidant capacity, *OSI* oxidative stress index, *MDA* malondialdehyde.

All-parameter comparisons were corrected for differences in age, pre-pregnancy BMI, gestational age at sampling, fasting Ins, fasting Glu, and HOMA-IR between the two subgroups, except for age, BMIs, and gestational age at sampling.

^†^ *P* < 0.05: compared with the high LDL-C subgroup in the control group.

^‡^ *P* < 0.05: compared with the medium LDL-C subgroup in the control group.

^§^ *P* < 0.05: compared with the high LDL-C subgroup in the GDM group.

^¶^ *P* < 0.05: compared with the medium LDL-C subgroup in the GDM group.

^a^ Plasma glucose levels during OGTT between 24 and 28 weeks of gestation.

^b^ Comparisons of fasting Ins, fasting Glu, and HOMA-IR were corrected for differences in age, pre-pregnancy BMI, and gestational age at sampling between the two groups.

^c^ Controls (high = 235, medium = 408, low = 176); GDM (high = 123, medium = 292, low = 156).

Similar patterns were observed in the control group. The low-LDL-C subgroup exhibited higher pre-pregnancy and delivery BMI, TG, and TG/HDL-C ratios, yet lower HDL-C, apoB, TC, non-HDL-C, AI, and apoB/apoA1 ratios than the high- and medium-LDL-C subgroups (*P* < 0.05); higher OGTT-fasting glucose levels than the medium-LDL-C subgroup (*P* = 0.041); and higher OGTT-1 h glucose levels (*P* = 0.049) and tended to have increased OGTT-fasting and 2 h glucose levels (*P* < 0.098) than the high-LDL-C subgroup. The medium-LDL-C subgroup also exhibited lower HDL-C, TC, non-HDL-C, AI, apoB, apoB/apoA1 ratios, and MDA levels than the high-LDL-C subgroup (*P* < 0.05, Table 3).

### Association of LDL-C levels with clinical and biochemical indicators

The Spearman’s correlation in women with GDM showed that LDL-C levels were positively correlated with apoB, TC, non-HDL-C, apoB/apoA1 ratios, HDL-C, AI, MDA, and apoA1 levels (*r* = 0.833, 0.807, 0.777, 0.583, 0.437, 0.296, 0.154, and 0.149, respectively; *P* < 0.001) and negatively correlated with TG/HDL-C ratios, pre-pregnancy BMI, OGTT-fasting glucose concentrations, and delivery BMI (*r* = − 0.267, − 0.147, − 0.144, and − 0.121, respectively; *P* < 0.01).

As illustrated in Table 4, a stepwise multivariate regression analysis demonstrated that apoB, TG, and apoA1 levels were key predictors of LDL-C levels in the GDM group (R^2^ = 0.718, adjusted R^2^ = 0.716, *P* < 0.001), and apoB, TG, apoA1, and OGTT-1 h glucose were significant predictors of LDL-C levels in all the participants (R^2^ = 0.719, adjusted R^2^ = 0.718, *P* < 0.001).

**Table 4:** Association of important independent variables with LDL-C levels by linear regression analysis in women with and without GDM.

	Unstandardized coefficients		Standardized coefficients	t	*P*
	B	SEM	β		
Model I: women with GDM were contained in the model					
Constant	-0.791	0.174		-4.538	< 0.001
ApoB (g/L)	3.398	0.099	0.858	34.294	< 0.001
ApoA1 (g/L)	0.317	0.058	0.131	5.455	< 0.001
TG (mmol/L)	-0.210	0.016	-0.327	-13.094	< 0.001
Model II: all participants were contained in the model					
Constant	-0.190	0.148		-1.284	0.200
ApoB (g/L)	3.475	0.068	0.854	50.957	< 0.001
ApoA1 (g/L)	0.228	0.041	0.090	5.535	< 0.001
TG (mmol/L)	-0.217	0.012	-0.312	-18.486	< 0.001
OGTT-1 h Glu (mmol/L)^a^	-0.042	0.009	-0.074	-4.501	< 0.001

The dependent variable is serum LDL-C levels.

*LDL-C* low-density lipoprotein cholesterol, *OGTT* oral glucose tolerance test, *TG* triglyceride, *ApoA* apolipoprotein A1, *ApoB* apolipoprotein B, *GDM* gestational diabetes mellitus.

^a^ OGTT-1 h glucose levels during between 24 and 28 weeks of gestation.

## Discussion

To our knowledge, this is the first study to explore the relationship between LDL-C level and the risk of GDM. Our findings indicated that decreased serum LDL-C levels were associated with an increased risk of GDM in the study population. Further analysis showed that the risk of GDM decreased by 17.6% per 1 mmol/L increase in serum LDL-C levels. The subgroup analysis revealed that reduced serum LDL-C levels were correlated with increased pre-pregnancy and delivery BMI, and unfavorable TG, HDL, and glucose metabolism in both the GDM and control groups. Moreover, apoB, TG, and apoA1 levels were significant predictors of serum LDL-C levels in women with GDM. Our study suggests that low serum LDL-C levels may be associated with the development of GDM.

Accumulating evidence indicates a potential link between dyslipidemia and abnormal glucose metabolism^9,15–18^. GDM, like T2DM, is characterized by reduced insulin sensitivity, impaired glucose tolerance, and dyslipidemia^1,9,19^. Studies suggest that an elevated risk of abnormal postpartum glucose metabolism in women with GDM is linked to increasing TG and LDL-C levels and decreasing TC levels during the mid-trimester of pregnancy^17,18^. In this study, a reduction in serum LDL-C levels during the third trimester of pregnancy or before delivery was associated with an elevated risk of GDM in pregnant women in Southwest China. Women with GDM and low LDL-C levels exhibited increased pre-pregnancy and delivery BMI, second-trimester OGTT-fasting glucose concentrations, and TG/HDL-C ratios, but reduced apoA1 and HDL-C levels, TC, non-HDL-C, AI, apoB, apoA1/apoB ratios, and MDA levels. Our findings indicate that although low LDL-C levels are linked to lower atherogenic lipoprotein concentrations, decreased LDL-C levels may contribute to an increase in pre-pregnancy and delivery BMI, and an adverse effect on glucose, TG, and HDL metabolism, thereby promoting the occurrence of GDM. This finding supports a previous study that showed that an elevated LDL-C trajectory during pregnancy can protect women with GDM against postpartum glucose intolerance^16^.

LDL, the primary transporter of serum cholesterol, transports cholesterol from endogenous and exogenous sources to peripheral cells^20,21^. Cholesterol is a vital constituent of biomembranes and acts as a precursor for many important biomolecules such as steroid hormones, bile acids, and vitamin D^18,20,22^. A compensatory increase in maternal serum cholesterol levels during pregnancy is necessary for the formation of placental hormones and fetal growth and development^18^. In contrast, LDLs are involved in the pathogenesis of atherosclerosis and coronary disease, leading to atherosclerotic cardiovascular diseases^20,21,23^. In this study, we demonstrated that lower LDL-C levels are associated with a higher incidence of GDM. However, the mechanism underlying the association between the risk of GDM and serum LDL-C levels remains unclear. Understanding and balancing the relationship between LDL-C levels and the occurrence of atherosclerosis and GDM requires further studies.

Serum LDL-C concentrations are dependent on the rates of production and clearance. Low-density lipoproteins are primarily formed by the degradation of circulating VLDL, which is the main carrier of fasting circulating TGs^24,25^. Therefore, increased VLDL generation and/or delayed conversion of VLDL to LDL could be critical factors contributing to elevated serum TG and reduced LDL-C levels. In this study, we found that women with GDM with low LDL-C levels tended to have higher TG levels than those with high LDL-C levels. Additionally, we demonstrated an inverse association between TG and LDL-C levels, with TG level identified as another significant predictor of LDL-C levels in the multivariate regression model. These findings suggest that women with GDM may have elevated VLDL production and/or reduced VLDL to LDL conversion.

Moreover, serum and cellular cholesterol balance is regulated by other multiple factors.

Insulin resistance is an important characteristic of GDM and related to increased VLDL particle size and decreased HDL and LDL particle sizes^26–29^. Women with GDM have a higher small dense LDL/large buoyant LDL particle ratio than women with normal glucose tolerance^27^. Moreover, an increased TG/HDL-C ratio has been linked to an elevation in small and dense LDL, which is vulnerable to oxidative stress and can be readily converted into oxidized LDL (ox-LDL)^20,24^. Small and dense LDL resulting from hypertriglyceridemia could enhance LDL-C transportation into cells by increasingits affinity for LDL receptor (LDLR)^30,31^. These findings suggest that an increased small LDL particle count or TG/HDL-C ratio may serve as a biomarker for GDM and severe insulin resistance.

Hormonal fluctuations further influence lipid metabolism. For example, women with GDM exhibit higher progesterone levels in the second trimester^32^, which enhances LDL oxidation in the placenta^33^. Additionally, lipoprotein lipase (LPL) plays a key role in accelerating TG-rich lipoprotein catabolism and facilitating transplacental transfer of TG-derived fatty acids^26,34^. LPL activity is enhanced by insulin and apoC2, and, on the contrary, attenuated by insulin resistance, apoC3, human placental lactogen, and angiopoietin-like protein 3, 4, and 8^26^. Reduced LPL activity profoundly affects the metabolism of TG-rich lipoproteins, causing an increase in TG levels and reduction in VLDL to LDL conversion. Therefore, impaired LPL activity may be involved in the occurrence and progression of abnormal lipid metabolism in women with GDM.

In pancreatic β-cells, cellular lipid and cholesterol availability may affect insulin production, storage, and secretion^30^. Similar to all cells, pancreatic β-cells can synthesize cholesterol through the mevalonate pathway. Concurrently, the uptake of LDL via LDLR is also a significant source of cellular cholesterol^30,31^. Inhibition of cholesterol biosynthesis may augment LDLR expression and cholesterol uptake by increasing the activation of sterol regulatory element-binding protein 2^30,31,35^. Conversely, HDL including apoA1 can remove excess cholesterol in β-cells through the reverse cholesterol transport process^35^. Oxidized lipoproteins, including ox-LDL, are transported into cells by scavenger receptors that remain unaffected by cellular cholesterol levels. Consequently, more cholesterol and oxidized lipids can enter the cells^20,36^. The accumulation of cholesterol and oxidized lipids in pancreatic β-cells has the potential to impair insulin production, storage, and secretion. This can lead to the degeneration of pancreatic islets and promote the occurrence and development of T2DM^30,31,37^.

The present study showed that women with GDM and low LDL-C levels had higher pre-pregnancy and delivery BMI, TG/HDL-C ratios, second-trimester OGTT-fasting glucose concentrations, and relatively high TG levels but lower apoA1 and HDL-C levels than those with medium and/or high LDL-C levels. These findings suggest that reduced LDL-C levels in women with GDM could be involved in the development of GDM through several mechanisms, including damaged metabolism of lipoproteins, enhanced cholesterol and oxidized lipid uptake, reduced the cellular cholesterol clearance, and impaired β-cells function. Further studies are required to clarify the underlying mechanism.

This study has certain limitations. First, we were unable to systematically measure serum lipid levels during pre-pregnancy, early pregnancy, and middle pregnancy, which could provide further evidence to support the link between serum LDL-C levels and susceptibility to GDM, and that it would be better if the risk for GDM development was estimated based on LDL-C levels before or at the beginning of pregnancy. Second, some participants were not evaluated for oxidative stress parameters because the sample volume was restricted or the samples contained bilirubin or hemolysis, which could have affected the statistical significance of the parameter analyses.

In conclusion, this study indicated that decreased serum LDL-C levels are associated with an elevated risk of GDM in Chinese women. The risk of GDM decreased by 17.6% per 1 mmol/L increase in serum LDL-C level. Subgroup analysis demonstrated a correlation between lower serum LDL-C concentrations and higher pre-pregnancy and delivery BMI, as well as unfavorable TG, HDL, and glucose metabolism. Our study suggests that stricter control of BMI and improved TG and HDL metabolism may be helpful in controlling the occurrence and development of GDM in women with low LDL-C levels. Our findings provide novel evidence on the role of lipid metabolism, particularly LDL metabolism, in the pathogenesis of GDM.

## Methods

### Study participants

This case-control study included 696 women with GDM and 1048 healthy pregnant women and was conducted at the Department of Obstetrics at the West China Second University Hospital between 2013 and 2021. Written informed consent was obtained from each participant before the commencement of the study. The study was conducted in accordance with the principles of the Declaration of Helsinki and approved by the hospital’s Institutional Review Board (No. 2017-033 for X Liu, No. 2020-036 for P Fan).

Each participant underwent an oral glucose tolerance test (OGTT) using a standard 75 g glucose solution between 24 and 28 weeks of gestation. According to the criteria for diagnosing GDM of the International Association of Diabetes and Pregnancy Study Groups^19^, a woman was diagnosed with GDM if she had one or more of the following results: fasting glucose ≥ 5.1 mmol/L, 1 h glucose ≥ 10.0 mmol/L, and/or 2 h glucose ≥ 8.5 mmol/L. Pregnant controls were concurrently recruited from the same hospital. The participants were excluded if they met any of the following conditions: pre-gestational diabetes, hypertensive disorder of pregnancy (including gestational hypertension, preeclampsia, or chronic hypertension), intrahepatic cholestasis of pregnancy, twin/multiple pregnancies, smoking, alcohol use, and other cardiac, hepatic, renal, autoimmune, or endocrine diseases. Women with previous GDM, premature deliveries, or who have undergone in vitro fertilization were excluded from the control group.

Clinical characteristics including maternal age, parity, gestational age, delivery and pre-pregnancy BMI (kg/m^2^), pregnancy weight gain, systolic and diastolic blood pressure (SBP and DBP), fasting, and 1 h- and 2 h-blood glucose concentrations of the OGTT in the mid-trimester, along with neonatal height and weight, were recorded. Macrosomia was defined as a neonatal birth weight ≥ 4000 g^5^.

Blood samples were collected in the third trimester or before delivery after at least 8 h of fasting. Plasma and serum were separated at 4 °C within 2 h and preserved at − 80 °C until analysis.

### Analysis of oxidative stress and glycolipid metabolic indicators

Plasma glucose, insulin, apoA1, apoB, TG, TC, LDL-C, HDL-C, serum MDA, total antioxidant capacity (TAC), and TOS were analyzed using methods described in the cited published articles^5,6,25,38^. The OSI, HOMA-IR, and atherosclerosis index (AI) were calculated as described in the cited published articles^25,39^. Intra- and inter-assay variations were < 5% and < 10%, respectively.

### Statistical analysis

Quantitative variables between the two groups and subgroups were compared using an independent sample *t*-test, one-way analysis of variance, nonparametric test (for variables with asymmetric distribution), or analysis of covariance (to correct for discrepancies in maternal age, pre-pregnancy BMI, gestational age at sampling, fasting insulin, fasting glucose, and HOMA-IR). The Chi-square (χ2) test was used to compare the differences in relative frequencies between the groups. The Spearman’s correlation analysis was used to assess the association between LDL-C levels and other indicators in women with GDM. Logistic regression was used to calculate odds ratios (OR) and 95% confidence intervals (CIs) to estimate the risk of GDM in relation to LDL-C levels. The effects of key factors (independent variables), including age, pre-pregnancy BMI, gestational age at sampling, pregnancy weight gain, SBP, DBP, gestational age, neonatal birth height and weight, OGTT-fasting glucose, OGTT-1 h glucose, OGTT-2 h glucose, fasting glucose, fasting insulin, apoA1, apoB, HDL-C, TG, TAC, TOS, and MDA, on LDL-C levels (dependent variable) were examined using multivariate stepwise regression analyses. We excluded some parameters, such as delivery BMI, HOMA-IR, TC, non-HDL-C, AI, TG/HDL-C ratio, apoB/apoA1 ratio, and OSI, in the multivariate regression analysis due to the presence of collinearity between them and some of the included independent variables. Statistical significance was set at a two-sided *P*-value of < 0.05. Statistical Program for Social Sciences (SPSS, version 21.0; IBM SPSS Statistics, IBM Corporation, Armonk, NY, USA) was used for all statistical analyses.

Statistical power was calculated using R version 4.0.2 (R Core Team, Vienna, Austria).

## Data Availability

Data that substantiate the conclusions of this study can be provided by the corresponding authors upon reasonable request.

## Abbreviations

AI: Atherosclerosis index
Apo: Apolipoprotein
BMI: Body mass index
CI: Confidence interval
DBP: Diastolic blood pressure
GDM: Gestational diabetes mellitus
HDL-C: High-density lipoprotein cholesterol
HOMA-IR: Homeostatic model assessment of insulin resistance
LDL-C: Low-density lipoprotein cholesterol
LDLR: Low-density lipoprotein receptor
MDA: Malondialdehyde
non-HDL-C: non-high-density lipoprotein cholesterol
OGTT: Oral glucose tolerance test
OR: Odds ratios
OSI: Oxidative stress index
SBP: Systolic blood pressure
T2DM: Type 2 diabetes mellitus
TAC: Total antioxidant capacity
TC: Total cholesterol
TG: Triglyceride
TOS: Total oxidant status
VLDL-C: Very low-density lipoprotein cholesterol
